# Non-invasive wireless electroencephalographic recording of the sleep–wake cycle in freely moving reptiles, birds, and mammals: a novel methodology compatible with animal welfare

**DOI:** 10.3389/fvets.2026.1736943

**Published:** 2026-02-13

**Authors:** Mario Fernández-Sánchez, Carlos Barros-García, Miguel Garzón

**Affiliations:** 1 Departamento de Anatomía, Histología y Neurociencia, Facultad de Medicina, Universidad Autónoma de Madrid, Madrid, Spain; 2 PhD Program in Neuroscience, Universidad Autónoma de Madrid – Cajal Institute, Madrid, Spain; 3 Department of Biology, Avanqua-Oceanogràfic S.L., Fundación Oceanogràfic, Valencia, Spain

**Keywords:** amniotes, animal welfare, behavioral monitoring, comparative neurophysiology, ethical neuroscience, non-invasive EEG, sleep–wake cycle, wireless monitoring

## Abstract

Animal welfare is increasingly assessed through the “Five Domains” framework, where monitoring brain activity via electroencephalography (EEG) is essential for objectively evaluating sleep–wake cycles and neurological health. However, traditional EEG studies in animals often require invasive procedures, anesthesia, and movement restriction, which compromise both animal welfare and natural behavior. To overcome these limitations, we developed a miniaturized wireless EEG device (24.8 × 24.8 × 8.2 mm; 5.2 g) with Bluetooth transmission, surface electrodes, and biocompatible adhesives. This system allows 10–12 h of recording without restricting movement while remaining compatible with animal welfare standards. We validated the methodology in three amniote species representing major vertebrate classes: Aldabra giant tortoise (*Aldabrachelys gigantea*), gentoo penguin (*Pygoscelis papua*), and aardvark (*Orycteropus afer*). Recordings were conducted on conscious, freely moving animals in zoological facilities, and signals were analyzed using spectral frequency analysis. Three distinct EEG patterns were consistently identified across all species: active wakefulness, characterized by desynchronized high-frequency waves (0–30 Hz) and locomotor activity; NREM sleep or a homologous state, marked by synchronized high-amplitude, low-frequency waves (0.5–4 Hz); and REM sleep or a homologous state, defined by desynchronized high-frequency waves without locomotor activity. Fundamental brainwave frequencies (delta, theta, alpha, beta and gamma) were consistent and conserved across species, while amplitude varied according to anatomical differences. Interestingly, we observed specific patterns of EEG frequencies distribution in the three species, reflecting unique evolutionary spectral profiles, such as alpha dominance during aardvark wakefulness, theta profusion in penguin wakefulness and REM sleep, and delta massiveness in tortoise NREM sleep. This non-invasive methodology successfully distinguishes and records sleep–wake patterns in reptiles, birds, and mammals without surgical procedures, demonstrating that high-quality neurophysiological data can be obtained while adhering to animal welfare principles. The system maintained signal integrity within a 15-meter range, allowing for naturalistic behaviors in home enclosures. The technique opens new possibilities for longitudinal behavioral studies, detection of neurological disorders, and comparative sleep research in captive animals, representing a significant advance toward more ethical practices in animal neuroscience.

## Introduction

Animal welfare is defined within a comprehensive framework of five domains that guide policies and actions aimed at ensuring a dignified and healthy life for all animals under human care and supervision (1, 2). These five domains promote beneficial practices in nutrition, environment, health, behavior, and mental state. Within this framework, the analysis of nervous system activity—particularly that of the brain—is essential for establishing appropriate practices and protocols that safeguard the welfare of animals under human management.

The study of brain electrical activity enables the transition from a subjective observation of animal behavior to an objective evaluation that informs research and interventions in areas such as abnormal behaviors, sleep–wake cycles, and pathologies including epilepsy. Of particular interest is the study of the sleep–wake cycle, which follows a circadian rhythm and is characterized by three general EEG patterns—wakefulness, NREM sleep, and REM sleep—across amniote species (reptiles, birds, and mammals) (3). Although the full range of sleep functions is not yet fully understood, there is strong evidence that adequate sleep duration and quality are fundamental to the welfare of all species, and thus represent a key element in animal welfare protocols. All physiological functions—cognitive, biochemical, and immune, among others—are negatively affected by inadequate sleep (4).

To understand the natural functioning of the nervous system in health, as well as its neurophysiology and associated features, a phylogenetic approach is necessary (5). This is because the nervous system is a conserved biological system across all branches of the animal kingdom, unified by a fundamental element: the neuro-electrical activity of neurons, whose oscillatory patterns are studied through electroencephalographic (EEG) techniques (6, 7).

Since the mid-20th century, EEG techniques have been widely applied in humans, mammals, and other phylogenetic groups to characterize brain physiology related to behaviors, pathologies, and especially the sleep–wake cycle (8). In humans, EEG recordings are commonly obtained with non-invasive electrodes placed on the scalp, as brain size and the thinness of overlying tissues allow the collection of high-quality electrical signals, provided the subject remains still and follows instructions. However, in other mammals, in birds, and in reptiles, EEG methods usually involve invasive procedures and/or obligatory movement restriction. The vast majority of animal EEG studies include prior anesthesia, surgery, and electrode implantation. This is largely due to the difficulty of conducting experiments in which the animal, moving freely with electrodes implanted and connected to polysomnography equipment, does not attempt to remove the setup, remains calm, and still exhibits normal behaviors, including sleep–wake cycles (9, 10). Although non-invasive electrophysiological recordings have been previously reported in non-human animals, for example in chicken (11, 12), mice (13, 14), rats (15, 16), cats (17, 18) and dogs (19, 20), these methods still require some degree of restriction or alteration of natural behavior, such as anesthesia, physical restraint or body-attached wires limiting movement.

Implanted electrodes—such as those used in electrocorticography or subdermal needle electrodes—have traditionally been used in behavioral, pathological, and sleep–wake cycle studies across various species. These methods require anesthesia and surgical interventions that not only prevent repeated continuous EEG recordings but also inevitably alter normal behavior (21). Currently available non-invasive EEG devices, designed primarily for humans, present limitations in size and electrode placement that hinder their application in many animal species.

This study focuses on the development and validation of a novel, non-invasive, wireless EEG methodology designed for continuous and stable detection of brain states in unanesthetized, freely moving amniotes. The primary aim of this study is to develop and implement a new EEG method for amniote species that enhances understanding of neuro-electrical characteristics, particularly those reflected in the sleep–wake cycle, but also in common behavioral patterns and pathological activity such as epilepsy. To achieve this, we developed and adapted cutting-edge technological devices that overcome the limitations of previous approaches while remaining compatible with the definition of animal welfare established by organizations responsible for managing the care of animals in zoos, aquariums, reserves, sanctuaries, and similar institutions. Likewise, this methodology can be applied in veterinary practice within agricultural facilities, equine stables, domestic animals, and sleep research laboratories.

## Methods

### Experimental subjects

We selected the following species for this study based on their phylogenetic divergence within the evolutionary tree, cranial size (allowing electrode placement in comparable positions), and ease of handling by caretakers:

Reptiles: *Aldabrachelys gigantea* (Aldabra giant tortoise), family Testudinidae, native to the Seychelles archipelago in the Indian Ocean.
Birds: *Pygoscelis papua* (gentoo penguin), family Spheniscidae, inhabiting sub-Antarctic islands.
Mammals: *Orycteropus afer* (aardvark), family Orycteropodidae, native to forested areas of central and southern Africa.

Table 1 summarizes basic information for the experimental cases, specifying the number of animals used, the number of recording sessions per subject, and the starting hours and recording duration in each species. A total of thirteen sessions were analyzed across six animals. Experimental protocols for all three species were approved by the respective animal ethics and welfare committees of the institutions where the animals resided: Oceanogràfic of Valencia and Fundación Parques Reunidos (Zoo of Madrid and Faunia).

**Table 1:** Experimental cases and recording parameters.

Species	Sex	Age (years)	Weight (Kg)	Number of recording sessions	Start time of recording	Recording duration
*Aldabrachelys gigantea* (*n* = 2)	Male	20	60	2	17:00–18:00 h	10–11 h
	Male	25	55	1		
*Pygoscelis papua* (*n* = 2)	Female	2	8	4	16:00–17:00 h	9–11 h
	Female	4	10	2		
*Orycteropus afer* (*n* = 2)	Female	5	42	3	17:00–18:00 h	8–10 h
	Male	5	37	1		

Summary of species, sex, age, and weight for all animals (*n* = 6). The table specifies the number of recording sessions conducted per subject, daily start time ranges (local time), and session duration. Thirteen total sessions were analyzed. Recording start times are noted in 24-h format (h). Species-specific durations reflect the continuous time elapsed from the start of the recording until completion.

### Experimental design and technological development

**Figure 1:**
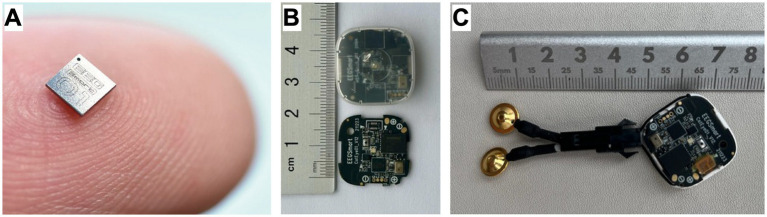
Experimental design and technological development. **(A)** Size of the new micro-electroencephalographic signal recording chip, EEGSmart01. **(B)** SmartEEG PCB with EEGSmart01 chip, encapsulated in the waterproof casing. **(C)** PCB reconfiguration with external electrode anchoring using JST connectors, length adapted to the skull of the studied species, cup-type termination, and new EEGSmart01 chip design.

The main challenge for non-invasive EEG acquisition was the design of a lightweight, autonomous device with sufficient battery capacity to record high-quality EEG signals for 8–12 h. In collaboration with SmartEEG®, we developed and adapted a next-generation EEG device through an electronic engineering process that included the design of new optimized chips for EEG recording, representing a technological advance for both human and animal applications (Figure 1).

#### Phase I

The first prototype was based on a SmartEEG Umindsleep PCB, adapted for animal recordings. Although the signal quality was acceptable, electrode placement and device size hindered proper application across animal species.

#### Phase II

The device underwent re-engineering to achieve higher signal precision than previous models, optimize interference and background noise, miniaturize the chip, and reduce power consumption (Figures 1A,B). The final PCB had the following specifications:

Name: UMindMirror (SmartEEG)
EEG performance: Input noise 4 μV; Common Mode Rejection Ratio (CMMR): 129
Size: 24.8 × 24.8 × 8.2 mm
Weight: 5.2 g
Nominal capacity: 50 mAh
Battery autonomy: 10–12 h using a lithium polymer (LiPo) battery

#### Phase III

Because the original electrode configuration was designed for humans, electrode connectivity was reconfigured with the following modifications (Figure 1C):

Development of an external connection through micro-soldering to the PCB.

Use of low-noise, biocompatible cables with JST connectors and anchors, with variable cable length depending on species.

Termination with gold-plated wet cup electrodes (Ag/AgCl, 10 mm diameter; Spes Medica), chosen for their stable electrochemical potential minimizing drift during 10–12 h recordings, low impedance allowing detection of weak signals through skin, feathers, or scales without invasive preparation, resistance to movement artifacts in active animals, and certified biocompatibility for prolonged skin contact—all essential for repeated non-invasive recordings across species.

### Recording specifications

#### EEG recording specifications

The EEG data were acquired using electrodes with the following specifications:

- Sampling Rate: 256 Hz.
- Resolution: 16-bit digital resolution (65,536 levels), covering a physical range of 3,643 μV. This provides a minimum detectable resolution of 0.0556 μV per level.
- Amplification & Gain: The system was calibrated with a gain of 0.0556 μV/level and a hardware offset of −454.47 μV. Signal conversion followed the formula:

𝑉𝑎(𝜇𝑉) = (𝐷𝑖𝑔𝑖𝑡𝑎𝑙𝑉𝑎𝑙𝑢𝑒×0.0556) − 454.47.

- Hardware Filters: A physical band-pass filter was applied between 0.5 Hz and 60 Hz, with a 50 Hz Notch filter to eliminate power line interference.

#### Locomotor activity tracking

Body movement was tracked using a triaxial accelerometer sampled at 2 Hz. Movement intensity was quantified using a three-step vector magnitude calculation:

- Axis differentiation: the cumulative change in acceleration for each axis (𝑥,𝑦,𝑧) was calculated as

$$d_{x}=\sum_{i=2}^{n}|x_{i}-x_{i-1}|$$

- Vector Combination: The total movement magnitude (𝑑) was determined by the Euclidean norm:

$$d=\sqrt{d_{x}^{2}+d_{y}^{2}+d_{z}^{2}}$$

- Normalization: The final “Movement Value” was scaled from 0 to 10 using a manufacturer-calibrated constant (11.9650815) to ensure comparability across subjects.

### Signal analysis and spectrogram parameters

Spectral analysis was performed using a Short-Time Fourier Transform (STFT) with the following parameters to ensure high frequency resolution:

- Windowing: a Kaiser window function was used to reduce spectral leakage.
- Segments & overlap: data were analyzed in 30-s segments composed of 2-s blocks with an 80% overlap between blocks.
- Scaling: power spectra were log-transformed (logarithmic scale: yes) to normalize the distribution of power across frequency bands.
- Artifact removal: periods exceeding the physical range of the amplifier (−2,276 μV to +1,367 μV) or containing non-physiological spikes identified via visual inspection were excluded from the final spectral averages.

### EEG recording protocol

**Figure 2:**
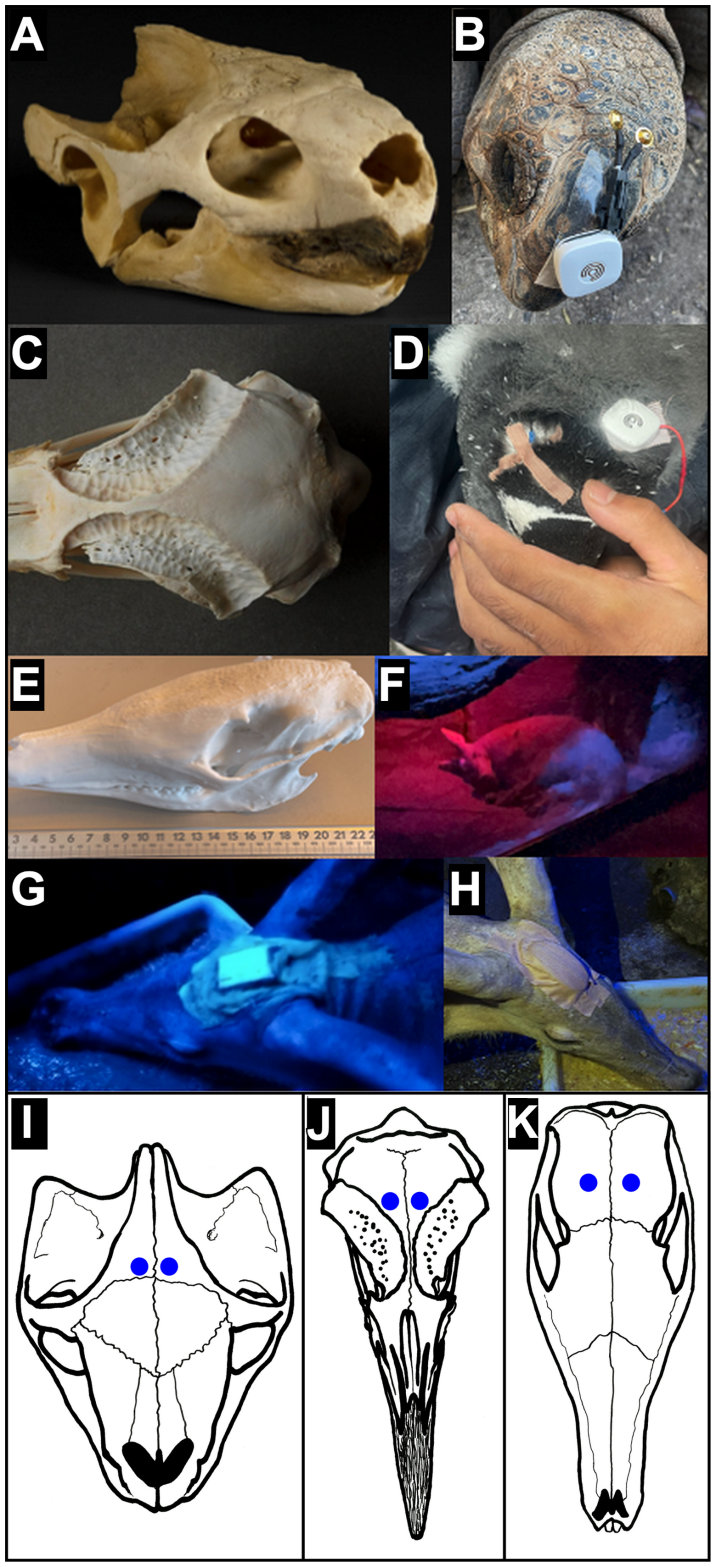
Skull preparation and electrode placement. **(A)** Tortoise (*Aldabrachelys gigantea*) skull. **(B)** Detail of the tortoise head with EEG sensor before electrode attachment. **(C)** Gentoo penguin (*Pygoscelis papua*) skull. **(D)** Detail of electrode fixation in a penguin using adhesive tape. **(E)** Aardvark (*Orycteropus afer*) skull. **(F–H)** Detail of electrode fixation in aardvarks using two-component silicone and adhesive tape. **(I)** Dorsal view of *Aldabrachelys gigantea* skull showing EEG electrodes position (blue dots). Illustration based on the 3D model of specimen MZB 2010–0257 from the Museu de Ciències Naturals de Barcelona, accessed via the Bioexplora 3D catalogue (https://www.bioexplora.cat/en). **(J)** Dorsal view of *Pygoscelis papua* (Gentoo penguin) skull showing EEG electrodes position (blue dots). Illustration based on photographies provided by Skullsite – Bird Skull Collection (https://skullsite.com). The original specimen is part of the collection managed by the Experimental Zoology Group, Wageningen University. **(K)** Dorsal view of *Orycteropus afer* skull showing EEG electrodes position. Illustration based on a 3D model of specimen UF Mamm 32,349 from the Florida Museum of Natural History, accessed via the iDigBio portal. 3D scanning and modeling provided by the oVert project (NSF DBI-1701714).

Instead of a standard electrode set, we manually configured two electrodes adapted to the cranial morphology of each species, ensuring easier placement over brain regions. Cup electrodes were modified by shortening the cable and micro-soldering them to the base connections of the UMindMirror PCB. Different attachment mechanisms were used depending on species (Figure 2):

- Tortoise: transparent double-sided 3 M adhesive tape, 2.5 cm wide (Galeno fabric, Martinez Llenas S. A.)
- Penguin: medical adhesive tape, skin colour, 1.5 cm wide (Galeno fabric, Martinez Llenas S. A.)
- Aardvark: medical adhesive tape, skin colour, 2.5 cm wide (Galeno fabric, Martinez Llenas S. A.) plus fast-setting two-component silicone (Body Double FAST, Smooth-On)

The device was always fitted on conscious animals, following animal welfare guidelines. Procedures were conducted within their enclosures with the assistance of regular caretakers to minimize stress. The protocol included:

1. Temporary separation of the animal for handling by keepers/veterinarians and device placement. When possible, this was done in the presence of conspecifics.
2. Cleaning of electrode contact areas with alcohol wipes and Nunprep® abrasive EEG gel. In penguins, down feathers were removed prior to placement.
3. Placement of the EEG device on the anterior cranial midline, anterior to the orbital cavity.
4. Positioning of electrodes coated with conductive/adhesive EEG paste (EEG-acp, CNSac Medshop GmbH, Germany) on both sides of the posterior cranium, over central regions of the hemisphere including the sensorimotor cortical area, followed by fixation with gauze soaked in collodion adhesive (NBN Medizin Elektronik GmbH, Germany).
5. Once fitted, animals could move freely within their enclosures with two restrictions: aquatic species could not enter the water, and all animals had to remain within 1–15 m of the Bluetooth-connected tablet/smartphone.
6. Animals could remain alone or with conspecifics, provided they did not attempt to remove the device.

### Electrodes placement

To ensure reproducibility of superficial EEG recordings across the three species, electrode placement over the central region of the cerebral hemisphere (sensorimotor cortex) was standardized using external cranial landmarks (Figure 2). In all specimens, the active electrode was positioned in a referential montage relative to the sagittal midline and the inter-aural axis (IAA).

Tortoise *(Aldabrachelys gigantea)*: Accounting for the specialized cranial shield, the electrode was positioned 5 mm lateral to the sagittal midline and 5 mm anterior to the IAA. The midpoint of the frontal plate served as the primary landmark to center recordings over the dorsal cerebral hemispheres, over the anterior part of parietal bones (45).

Penguin *(Pygoscelis papua)*: The electrode was placed 12 mm lateral to the sagittal midline and 10 mm anterior to the IAA. This location aligns with the posterior margin of the orbital rim, overlying the frontal bone to capture EEG activity while avoiding the supraorbital glands (44).

Aardvark *(Orycteropus afer)*: Given its dolichocephalic morphology, the electrode was placed over the parietal bone, 15 mm lateral to the sagittal midline and 10 mm anterior to the IAA. This targets the post-orbital region, minimizing artifacts from the temporal musculature (43).

### Connection, signal check, and recording initiation

After placement, the EEG device was connected via Bluetooth to the freely available Android Sleep Recorder 1.2.0 Pro app, installed on a tablet within a 15 m line-of-sight range. Signal quality, electrode placement, and battery status were verified before starting the recording session.

### Recording process and data storage

EEG data were transmitted via Bluetooth and stored on the tablet within the directory structure of the Sleep Recorder app. The steps required for proper recording were:

- Create an individual profile adapted to the species.
- Connect the UMindMirror device via Bluetooth.
- Test signal quality.
- Start and end the recording session.

Data were saved in European Data Format (EDF), an open, non-proprietary standard for medical time-series data, ensuring interoperability and compatibility with different analysis platforms and facilitating data sharing between institutions.

### EEG signal analysis

EEG signals were analyzed using the open-source, cross-platform software EDFbrowser (https://www.teuniz.net/edfbrowser/). To characterize the electroencephalographic (EEG) profile across different behavioral states, we calculated the relative power of the standard frequency bands (delta, theta, alpha, beta, and gamma) as a percentage of the total spectral power. For each species (tortoise, penguin, and aardvark), we analyzed five 30-s epochs obtained from representative episodes of each state (Wakefulness, NREM, and REM sleep). Statistical comparisons of these relative power values were performed using a nested repeated-measures ANOVA (StatView 5.0, SAS Institute Inc., Cary, USA). This design accounted for the hierarchical structure of the data by nesting multiple recordings within individual subjects. By partitioning the total variance into within-subject and between-subject components, this approach avoided pseudoreplication and ensured that group comparisons were based on the appropriate level of replication. Post-hoc analyses were conducted using Tukey’s Honest Significant Difference (HSD) test to identify specific pairwise differences between states and species, with the threshold for statistical significance set at *p* ≤ 0.05.

## Results

We successfully implemented the non-invasive EEG recording methodology in all three studied species belonging to the three Classes of amniotes: reptiles (*Aldabrachelys gigantea*), birds (*Pygoscelis papua*), and mammals (*Orycteropus afer*). In each case, we obtained stable signals of sufficient quality to identify and differentiate the main brain states: active wakefulness, NREM sleep, and REM sleep.

### General findings across species

As per standard nomenclature in sleep and arousal research, desynchronized activity refers to a brain state where the EEG displays low-amplitude, mixed-frequency patterns. In all three species, three distinct EEG patterns were consistently observed based on synchronization-desynchronization features present in the recordings:

1. Wakefulness – characterized by desynchronized, low-amplitude mixed-frequency activity dominated either by beta (12–30 Hz), typically associated with locomotor activity or alertness, or alpha (8–12 Hz) when relaxed with eyes closed.
2. NREM Sleep (or homologous state) – characterized by synchronized lower-frequency activity, with high-amplitude, overtly predominated by delta waves (0.5–4 Hz) in the deeper NREM episodes, and associated with motor rest.
3. REM Sleep (or homologous state) – characterized by desynchronized, low amplitude mixed-frequency activity resembling wakefulness (“paradoxical sleep”), with characteristic activity in the theta band (4–8 Hz) but without locomotor activity.

To ensure the accuracy of these observations, we utilized continuous video monitoring during the study. A comparative analysis of this footage confirmed that the animals’ behavioral parameters remained consistent with baseline periods when the device was not present. Furthermore, since recordings were primarily conducted during habitual rest periods, the video data verified that sleep patterns were undisturbed and occurred naturally as on non-recordin days.

Spectral analysis confirmed the presence of delta (0.5–4 Hz), theta (4–8 Hz), alpha (8–12 Hz), and beta (12–30 Hz) bands across all species, indicating that the frequency ranges of brain oscillations are conserved among amniotes. However, signal amplitude varied between species, largely reflecting anatomical differences such as skull thickness, tissue composition, and electrode placement.

### Species-specific results

#### *Aldabrachelys gigantea* (Aldabra giant tortoise)

**Figure 3:**
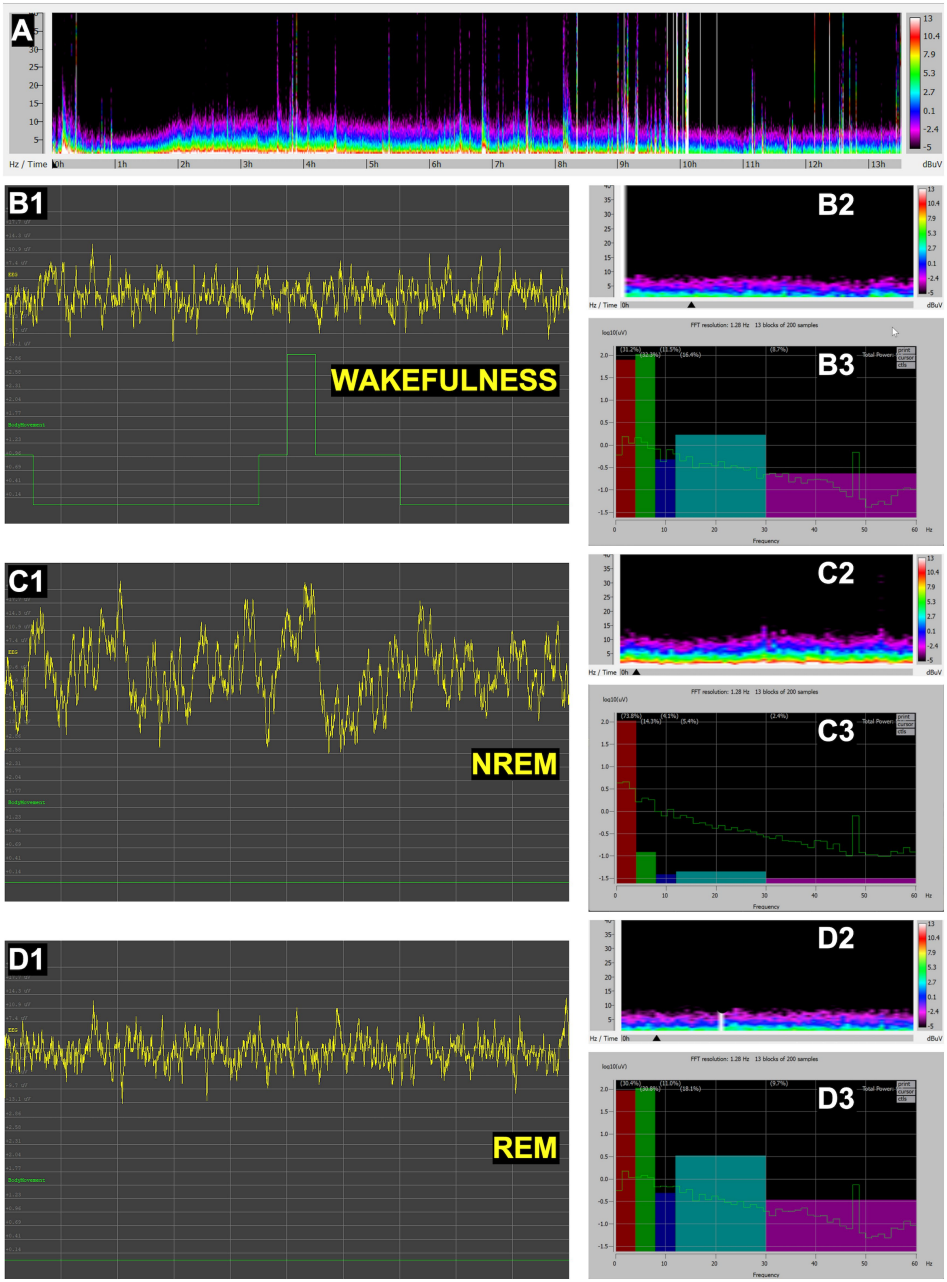
EEG recordings in *Aldabrachelys* (*Aldabrachelys gigantea*). **(A)** Full spectral density matrix of a 13-h recording, showing initial wakefulness in the first 2 h, followed by NREM-like sleep with slow waves and higher amplitude gradually decreasing until hour 10, when wakefulness predominates. Representative 10-s fragments of wakefulness **(B1)**, NREM sleep **(C1)**, and REM sleep **(D1)**, showing EEG channel (yellow) and movement (green); 30-min spectral density with black arrow indicating the spectrogram region corresponding to the 10-s EEG fragment shown, respesctively, in **B1, C1** and **D1** (**B2,C2,D2**); and power spectrum of the same 10-s fragment (thin green line), expressed also as relative power in delta (0.5–4 Hz), theta (4–8 Hz), alpha (8–12 Hz), beta (12–30 Hz), and gamma (30–60 Hz) bands, represented, respectively, in a color histogram (**B3,C3,D3**). In panels **B3, C3** and **D3**, the green lines illustrate the power spectral density for each state (**B3**, wakefulness; **C3**, NREM sleep; **D3**, REM sleep), plotted on a logarithmic *Y*-axis to allow for the visualization of power across multiple orders of magnitude. Overlaid color bars (histograms) represent the mean relative power, defined as the percentage of power in a specific band relative to the total integrated power of the full spectrum. Compared to **C3**, the marked reduction in high-voltage (delta) slow oscillations in **B3** and **D3** denotes a remarkable shift to a desynchronized cortical state during wakefulness and REM. Note on scaling: due to the dual-scaling of these panels **B3, C3** and **D3**, the heights of the histogram bars correspond to a linear percentage scale (values provided in parentheses above each bar) and are not mapped to the logarithmic *Y*-axis. The bar height reflects the total integrated area under the curve for that specific frequency range, which may visually differ from peak amplitude due to the logarithmic compression of the green spectral lines.

Recordings in tortoises revealed clear transitions between wakefulness and sleep states (Figure 3). Wakefulness was associated with irregular, low-amplitude, mixed-frequency activity (Figures 3B1,B3). During NREM sleep, the EEG prominently exhibited slow-wave activity within the delta frequencies (0.5–4 Hz) with high amplitude (Figures 3C1,C3). A REM-like state was identified (Figures 3D1,D3); it showed desynchronized, mixed-frequency activity (Figure 3D3) quite similar to that observed in wakefulness, but without movement (Figure 3D1), and thus clearly distinct from active wakefulness. These findings confirm the presence of sleep states homologous to those described in other reptiles.

#### *Pygoscelis papua* (gentoo penguin)

**Figure 4:**
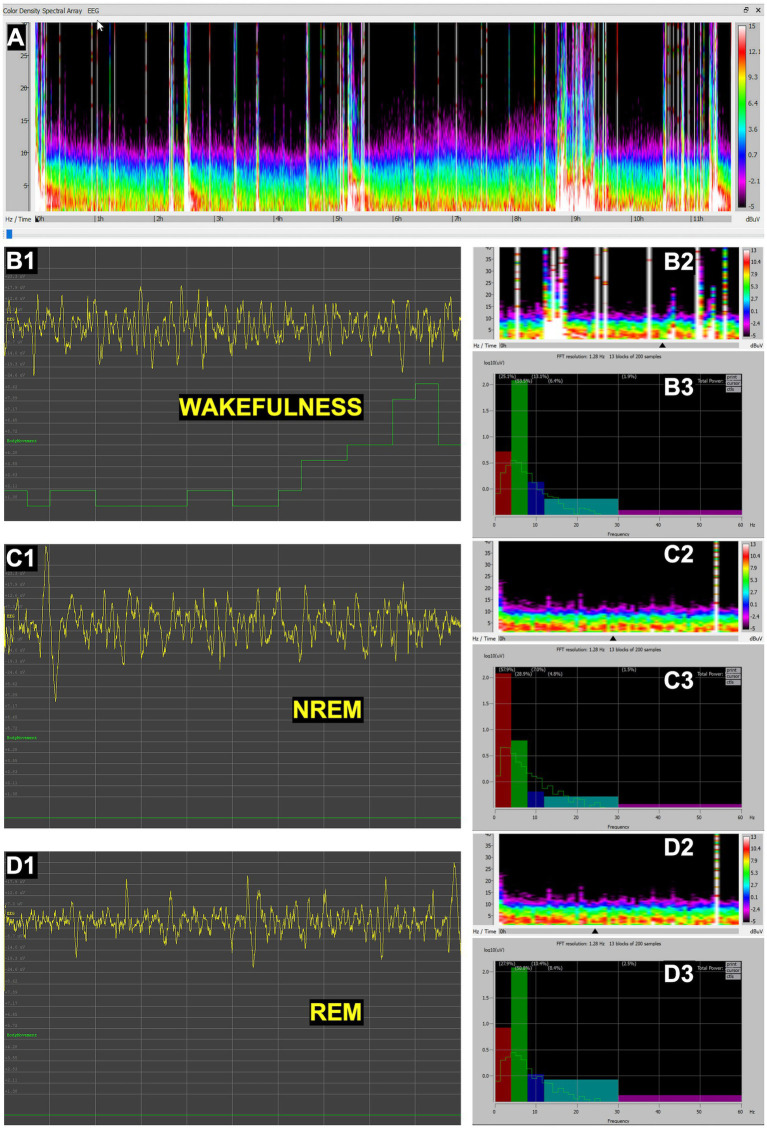
EEG recordings in Gentoo penguin (*Pygoscelis papua*). **(A)** Full spectral density matrix of a 12-h recording, showing alternating short periods of NREM and REM sleep. Representative 10-s fragments of wakefulness **(B1)**, NREM sleep **(C1)**, and REM sleep **(D1)**, showing EEG channel (yellow) and movement (green); 30-min spectral density with black arrow indicating, respectively, the region corresponding to the 10-s EEG fragment shown in **B1, C1** and **D1** (**B2,C2,D2**); and power spectrum of the same 10-s fragment (thin green line), expressed also as relative power in delta (0.5–4 Hz), theta (4–8 Hz), alpha (8–12 Hz), beta (12–30 Hz), and gamma (30–60 Hz) bands, represented, respectively, in a color histogram (**B3,C3,D3**). Note that the NREM **(C1)** and REM **(D1)** fragments shown are very close in time **(C2,D2)**. In panels **B3, C3** and **D3**, the green lines illustrate the power spectral density for each state (**B3**, wakefulness; **C3**, NREM sleep; **D3**, REM sleep), plotted on a logarithmic *Y*-axis to allow for the visualization of power across multiple orders of magnitude. Overlaid color bars (histograms) represent the mean relative power, defined as the percentage of power in a specific band relative to the total integrated power of the full spectrum. Note on scaling: Due to the dual-scaling of these panels **B3, C3** and **D3**, the heights of the histogram bars correspond to a linear percentage scale (values provided in parentheses above each bar) and are not mapped to the logarithmic *Y*-axis. The bar height reflects the total integrated area under the curve for that specific frequency range, which may visually differ from peak amplitude due to the logarithmic compression of the green spectral lines.

In penguins, EEG recordings showed well-defined wake and sleep cycles (Figure 4). Wakefulness was characterized by desynchronized activity during locomotion and environmental exploration (Figures 4B1,B3). NREM sleep exhibited slow, high-amplitude oscillations with predonant power in the delta waves band (Figures 4C1,C3), while REM sleep was distinguished by mixed desynchronized activity mainly in the theta band (Figure 4D3), occurring in the absence of movement (Figure 4D1). Periods of REM activity were shorter and more fragmented compared with mammals, and were easily detectable alternating with NREM sleep in the spectrograms (Figures 4C2,D2), consistent with previous reports in birds. These results demonstrate the capacity of the methodology to capture avian sleep states in a non-invasive manner.

#### *Orycteropus afer* (aardvark)

**Figure 5:**
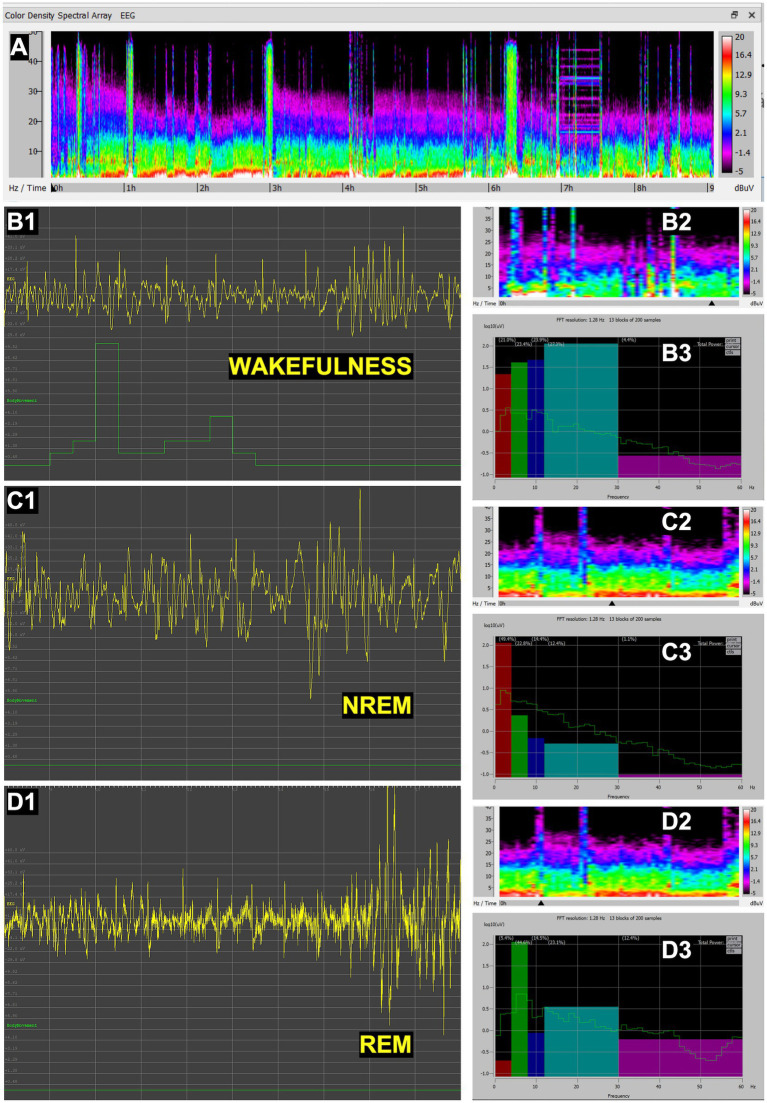
EEG recordings in aardvark (*Orycteropus afer*). **(A)** Full spectral density matrix of a 9-h recording, showing initial wakefulness and onset of NREM sleep with slow waves interspersed with brief periods of synchronized low-amplitude REM sleep. Representative 10-s fragments of wakefulness **(B1)**, NREM sleep **(C1)**, and REM sleep **(D1)**, showing EEG channel (yellow) and movement (green); 30-min spectral density with black arrow indicating the spectrogram region corresponding to the 10-s EEG fragment shown, respectively, in **B1, C1** and **D1** (**B2,C2,D2**); and power spectrum of the same 10-s fragment (thin green line), expressed also as relative power in delta (0.5–4 Hz), theta (4–8 Hz), alpha (8–12 Hz), beta (12–30 Hz), and gamma (30–60 Hz) bands, represented, respectively, in a color histogram (**B3,C3,D3**). In panels **B3, C3** and **D3**, the green lines illustrate the power spectral density for each state (**B3**, wakefulness; **C3**, NREM sleep; **D3**, REM sleep), plotted on a logarithmic *Y*-axis to allow for the visualization of power across multiple orders of magnitude. Overlaid color bars (histograms) represent the mean relative power, defined as the percentage of power in a specific band relative to the total integrated power of the full spectrum. Note on scaling: due to the dual-scaling of these panels **B3, C3** and **D3**, the heights of the histogram bars correspond to a linear percentage scale (values provided in parentheses above each bar) and are not mapped to the logarithmic *Y*-axis. The bar height reflects the total integrated area under the curve for that specific frequency range, which may visually differ from peak amplitude due to the logarithmic compression of the green spectral lines.

In aardvarks, EEG recordings also revealed the three primary sleep–wake cycle states (Figure 5). Wakefulness showed low-amplitude, mixed-frequency activity (Figures 5B1,B2), which was especially pronounced in the beta band during locomotion and exploratory behaviors (Figure 5B3). NREM sleep presented as salient synchronized, high-amplitude delta waves over a faint mixed frequency background (Figure 5C1). In contrast, REM sleep displayed desynchronized, mixed-frequency activity (Figure 5D) showing a rather noticeable theta peak (Figures 5D1,D3), without any trace locomotor activity (Figure 5D1). The EEG patterns were comparable to those described in other mammals, validating the methodology for large-bodied terrestrial mammals.

### Signal quality and methodological performance

The non-invasive system used in this study maintained stable recordings for 10–12 h, covering complete sleep–wake cycles. Bluetooth transmission was effective within a round perimeter up to 15 m radius, allowing naturalistic observation of the animals in their enclosures.

The use of wet cup electrodes (Ag/AgCl) provided low impedance and minimized motion artifacts, even in active animals. Minor variations in amplitude across species were attributable to anatomical differences rather than methodological limitations.

### Frequency bands EEG power analysis

To assess the significance of EEG frequency band power densities of the different behavioral vigilance states across species, we designed a nested three-way ANOVA (factors: species, state, frequency band) evaluating whether there was significant variability in relative EEG power with respect to (1) species, (2) state and/or (3) frequency band. The overall test revealed a significant three-way interaction between species, state and frequency band (F_16,96_ = 31.41, *p* < 0.0001), indicating that the distribution of spectral power across different frequency bands during sleep–wake states varies fundamentally between the tortoise, penguin and aardvark. Significant two-way interactions were also found for state x frequency band (F_8,96_ = 523.64, *p* < 0.0001) and species x frequency band (F_8,48_ = 210.24, *p* < 0.0001). Given the significant three-way interaction, subsequent analyses were decomposed by species to examine the specific state-dependent spectral shifts within each group.

#### EEG spectral profile of the tortoise

**Figure 6:**
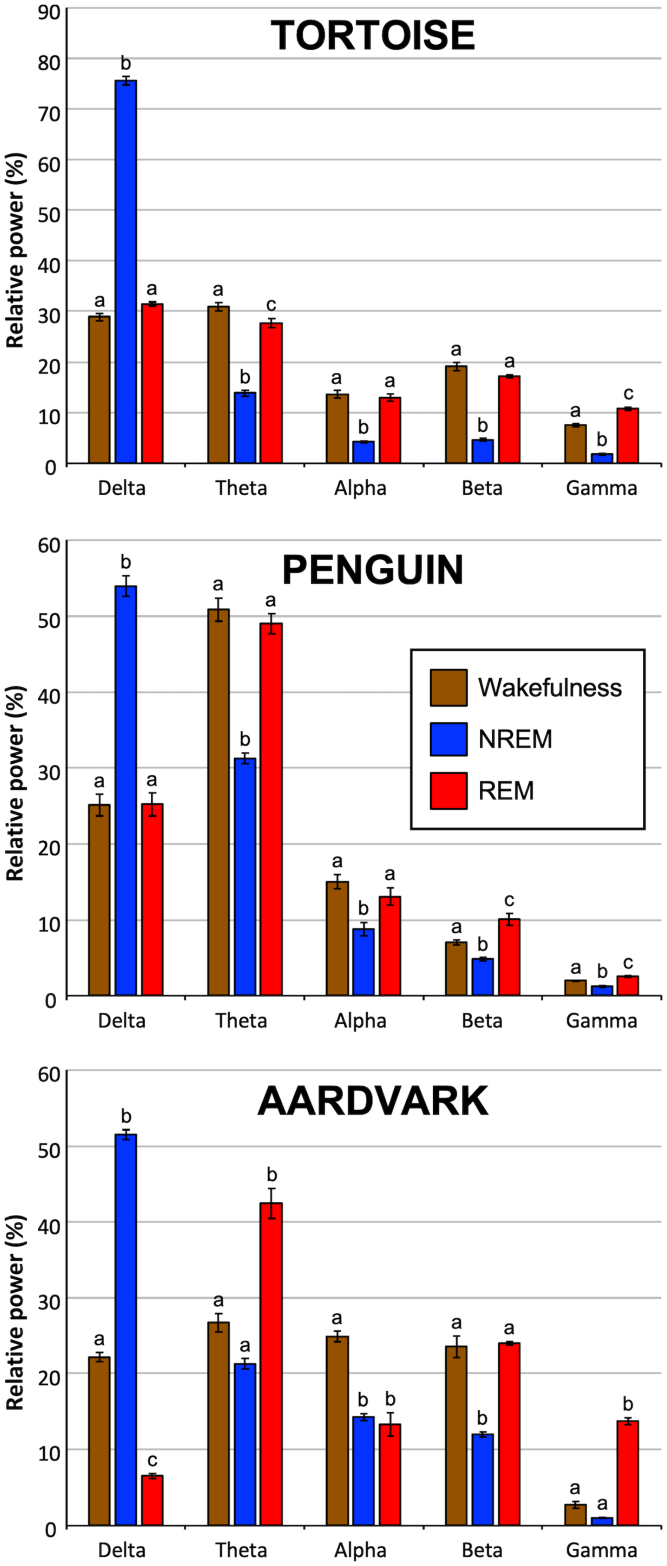
Relative EEG power densities across vigilance states in the three amniote species. Normalized power values for specific frequency bands were compared among wakefulness, NREM, and REM sleep states for tortoise, penguin, and aardvark (one-way ANOVAs for repeated measures). Each bar represents the mean percentage value ± SEM of each band relative to total power (0.5–60 Hz) obtained from 30-s epochs from fragments in consecutive episodes of wakefulness, NREM, and REM sleep (*n* = 5). Within each frequency band and species block, states sharing a common letter are not significantly different (*p* > 0.05), while those with no shared letters differ significantly (*p* ≤ 0.05) according to Tukey’s Honestly Significant Difference post-hoc test.

Within the tortoise group, a two-way repeated measures ANOVA revealed a significant state x frequency band interaction (F_8,32_ = 521.98; *p* < 0.0001), confirming that spectral distribution was dependent on the animal’s vigilance state. To further investigate this interaction, simple main effects were analyzed, revealing significant variations across all frequency bands. Post-hoc comparisons showed that delta relative power was significantly higher during NREM sleep compared to both wakefulness and REM sleep (F_2,8_ = 2286.96; *p* < 0.0001). Conversely, in all remaining frequency bands relative power in wakefulness and REM sleep was significantly higher than in NREM sleep (theta: F_2,8_ = 351.78; *p* < 0.0001; alpha: F_2,8_ = 97.1; *p* < 0.0001; beta: F_2,8_ = 185.05; *p* < 0.0001; gamma: F_2,8_ = 201.92; *p* < 0.0001). Specifically, in theta band, power during wakefulness was significantly higher than in REM sleep, whereas the opposite pattern was observed in the gamma band (Figure 6).

#### EEG spectral profile of the penguin

Regarding the penguin, the two-way repeated measures ANOVA evidenced a significant state x frequency band interaction (F_8,32_ = 85.57; *p* < 0.0001). Consequently, separate one-way ANOVAs were conducted for each band to assess the effect of state on relative power. In the delta band, power was significantly higher during NREM sleep than in wakefulness and REM sleep (F_2,8_ = 108.18; *p* < 0.0001). On the contrary, for all other bands NREM sleep relative power was significantly lower than in wakefulness and REM sleep (theta: F_2,8_ = 87.13; *p* < 0.0001; alpha: F_2,8_ = 12.63; *p* < 0.0001; beta: F_2,8_ = 25.18; *p* < 0.0001; gamma: F_2,8_ = 32.33; *p* < 0.0001). Finally, post-hoc comparisons revealed that in the beta and gamma bands, relative power during REM sleep was significantly higher than in wakefulness (Figure 6).

#### EEG spectral profile of the aardvark

Regarding the aardvark, the two-way repeated measures ANOVA also revealed a significant state x frequency band interaction (F_8,32_ = 189.57; *p* < 0.0001). Subsequent one-way ANOVAs showed that delta band relative power was significantly higher during NREM sleep than in both wakefulness and REM sleep (F_2,8_ = 1892.98; *p* < 0.0001). Conversely, in the theta, beta and gamma bands, NREM power was significantly lower than in wakefulness and REM sleep (theta: F_2,8_ = 48.02; *p* < 0.0001; beta: F_2,8_ = 53.68; *p* < 0.0001; gamma: F_2,8_ = 258.37; *p* < 0.0001). Specifically, within the theta and gamma bands, relative power during REM sleep was significantly higher than in wakefulness. Finally, in the alpha band, relative power in both NREM and REM sleep was significantly lower than that recorded during wakefulness (F_2,8_ = 63.85; *p* < 0.0001) (Figure 6).

#### Comparison of EEG power distribution between species

Finally, a global analysis was performed to compare spectral characteristics between species. Two-way ANOVAs for repeated measures (factors: species x frequency band) revealed significant changes in relative EEG power among species for the three behavioral vigilance states (wakefulness: F_8,48_ = 415.05; *p* < 0.0001; NREM: F_8,48_ = 158.82; *p* < 0.0001; REM: F_8,48_ = 79.19; *p* < 0.0001). Subsequent one-way repeated measures ANOVAs for each band were then utilized to assess simple effects and identify state-dependent variations through post-hoc comparisons, as shown in Table 2.

**Table 2:** Relative EEG power densities of tortoise, penguin, and aardvark species across sleep-wakefulness cycle states.

State	Band	Tortoise	Penguin	Aardvark	F_2,8_	p≤
WAKEFULNESS	Delta	28.88 ± 0.73	25.12 ± 1.40	22.14 ± 0.59*	10.41	0.0059
	Theta	30.92 ± 0.79	50.90 ± 1.50*	26.72 ± 1.22^#^	97.06	0.0001
	Alpha	13.60 ± 0.75	15.02 ± 0.95	24.90 ± 0.72*^,#^	56.06	0.0001
	Beta	19.10 ± 0.75	7.02 ± 0.34*	23.52 ± 1.43^#^	55.65	0.0001
	Gamma	7.50 ± 0.27	1.94 ± 0.10*	2.72 ± 0.44*	142.93	0.0001
NREM	Delta	75.62 ± 0.82	53.96 ± 1.37*	51.48 ± 0.67*	123.64	0.0001
	Theta	13.82 ± 0.55	31.28 ± 0.75*	21.30 ± 0.67*^,#^	126.50	0.0001
	Alpha	4.18 ± 0.17	8.74 ± 0.90*	14.30 ± 0.45*^,#^	76.39	0.0001
	Beta	4.58 ± 0.21	4.84 ± 0.20	11.94 ± 0.34*^,#^	326.82	0.0001
	Gamma	1.80 ± 0.20	1.18 ± 0.11*	0.98 ± 0.07*	10.67	0.0055
REM	Delta	31.44 ± 0.43	25.24 ± 1.51*	6.54 ± 0.35*^,#^	194.73	0.0001
	Theta	27.68 ± 0.86	49.06 ± 1.30*	42.40 ± 1.98*^,#^	47.32	0.0001
	Alpha	12.94 ± 0.72	13.06 ± 1.13	13.30 ± 1.49	0.022	0.978
	Beta	17.14 ± 0.34	10.10 ± 0.76*	24.00 ± 0.20*^,#^	177.68	0.0001
	Gamma	10.80 ± 0.33	2.54 ± 0.14*	13.76 ± 0.44*^,#^	298.09	0.0001

Mean relative power values and standard errors (in percentage of the total EEG power −0.5–60.0 Hz-) for the delta, theta, alpha, beta and gamma EEG bands obtained from 30-s EEG fragments during episodes of wakefulness, NREM and REM states. Values obtained from the statistical comparisons (one-way ANOVAs for repeated measures) among the three species under study (tortoise, penguin and aardvark) are also provided. *Statistically significant differences with respect to tortoise values, Tukey’s Honestly Significant Difference post-hoc test; ^#^Statistically significant differences with respect to penguin values, Tukey’s Honestly Significant Difference post-hoc test.

Briefly, during wakefulness, the penguin exhibited a markedly higher theta power compared to the other species, while the aardvark was characterized by a significantly higher alpha relative power. In contrast, the tortoise showed the highest levels of gamma power during this state (Table 2). During NREM sleep, although all species showed a delta-dominant profile (Figure 6), the tortoise reached significantly higher delta levels compared to the penguin and the aardvark. The aardvark maintained significantly higher power in the alpha and beta bands during this stage compared to the other two species (Table 2). Finally, in REM sleep, striking differences were observed in the delta component and high-frequency range. The aardvark showed a marked suppression of delta power, which was significantly lower than in the tortoise and penguin. Regarding fast oscillations, the aardvark exhibited the highest beta power, while gamma power reached its pead in the aardvark and tortoise, both being significantly higher than the levels observed in the penguin. Notably, alpha power during REM sleep was the only parameter that remained statistically indistinguishable across the three species (Table 2).

### Summary of results

Overall, this methodology enabled the identification of conserved sleep–wake states (wakefulness, NREM, and REM) in reptiles, birds, and mammals. Spectral frequency analysis demonstrated that the distribution of EEG frequency bands (delta, theta, alpha, beta) is consistent across these groups, supporting the evolutionary conservation of sleep mechanisms. However, species-specific EEG patterns were also overtly detected. The system proved effective, reproducible, and compatible with animal welfare standards, confirming its potential for future applications in comparative neuroscience, veterinary medicine, and zoological research.

## Discussion

The present study demonstrates the feasibility of a novel non-invasive and wireless EEG methodology for recording brain activity in unanesthetized and freely moving individuals of three phylogenetically distinct amniote species belonging, respectively, to reptiles, birds, and mammals. The implementation of this technique allowed us to identify and differentiate the fundamental states of wakefulness, NREM sleep, and REM sleep in *Aldabrachelys gigantea* (tortoise), *Pygoscelis papua* (penguin), and *Orycteropus afer* (aardvark).

### Methodological advances and contributions

Traditional EEG studies in non-human animals have relied primarily on invasive methods requiring anesthesia, surgical electrode implantation, and/or physical restraint, procedures that inevitably compromise animal welfare and alter normal behaviors (9, 21). In contrast, the present methodology is based on a lightweight, autonomous device coupled with wet Ag/AgCl cup electrodes, providing stable, long-duration recordings without the need for invasive preparation or prolonged immobilization.

This approach represents a significant advancement in both comparative neuroscience and animal welfare, as it aligns with the five-domain model of welfare (1, 2), enabling neurophysiological monitoring without compromising behavioral integrity. The ability to obtain 10–12 h of continuous EEG data under naturalistic conditions opens new opportunities for longitudinal studies in zoological institutions, veterinary practice, and wildlife conservation programs.

### Comparative neurobiology of sleep

Our results provide evidence of conserved sleep–wake dynamics across amniotes. In all three species, wakefulness was marked by low-amplitude, high-frequency activity; NREM sleep by synchronized, high-amplitude delta waves; and REM sleep by desynchronized, high-frequency activity without locomotion. These findings are consistent with previous descriptions of sleep in reptiles, birds, and mammals (3, 22, 23).

The identification of REM-like states in *Aldabrachelys gigantea* further supports the growing body of evidence that reptiles exhibit homologous sleep states to those described in birds and mammals, reinforcing the hypothesis of an ancient evolutionary origin of REM sleep (24, 25). In the homeothermic species examined (gentoo penguin and aardvark), the observed NREM sleep patterns, characterized by high-amplitude delta waves, and REM sleep, characterized by low-voltage desynchronized activity, are consistent with established descriptions in avian and mammalian literature (3, 8). The fragmented nature of REM sleep in penguins is in line with avian-specific adaptations, likely related to ecological pressures such as predation risk (26). The aardvark recordings, meanwhile, exhibited patterns analogous to those of other large terrestrial mammals, validating the method’s applicability in diverse mammalian taxa.

The electrophysiological profile of the aardvark provides a compelling illustration of the mammalian sleep state. Our findings align with comparative data indicating that mammals exhibit a high-frequency, low-amplitude EEG during REM sleep, characterized by a notable concentration of power in the beta and gamma bands (27, 28, 30, 31). This high-frequency power is significantly more pronounced in the aardvark than in non-mammalian amniotes such as the tortoise or the penguin, where active (REM) sleep states often lack the high-spectral intensity found in eutherian mammals (25). This reinforces the view that robust cortical activation during REM is a hallmark of the mammalian lineage. Also, a primary finding in the aardvark was the significantly higher alpha power during wakefulness compared to the other species. In mammals, alpha oscillations are recognized as essential for top-down inhibitory control and orchestrating long-range cortical communication (29). This alpha dominance, which was significantly suppressed during both NREM and REM sleep in our subjects, suggests a high degree of cortical integration during arousal, consistent with models of synaptic homeostasis where the transition to NREM serves to downscale the high-energy synaptic activity built up during alpha-dominant wakefulness (32). The specific suppression of alpha during sleep reinforces the theory that mammalian sleep requires a fundamental reorganization of the thalamocortical loops that sustain waking consciousness (24).

The comparison between the tortoise and the penguin offers a unique window into the evolution of sleep from the common stem-amniote ancestor. While both species showed state-dependent modulations, the tortoise exhibited a marked spectral homogeneity in REM, with delta levels significantly higher than those of the other species. This suggests that reptilian NREM sleep might represent a less differentiated and highly stable state compared to the more complex spectral distributions seen in birds and mammals (33). In contrast, the penguin displayed a shift towards faster oscillatory dynamics, with significant theta dominance during wakefulness and REM sleep. This shift likely supports the metabolic and cognitive demands of endothermy—where high-frequency neural activity facilitates the complex cognitive maps and efficient foraging necessary to sustain high metabolic rates (34). Furthermore, the specific dominance of theta oscillations is increasingly recognized as a requirement for complex navigation, particularly for encoding spatial trajectories and managing high-level information processing in avian species (36).

The successful detection of sleep patterns in such different animals using non-invasive surface electrodes supports the sensitivity and reliability of our methodology, indicating that anatomical differences—such as the presence of feathers, skull thickness, and subcutaneous fat—do not impede the acquisition of interpretable EEG signals when appropriate recording technology is employed. These findings validate the feasibility of non-invasive neurophysiological monitoring across phylogenetically distant taxa and establish a methodological framework applicable to both research and clinical contexts.

### Implications for animal welfare and management

The capacity to record sleep and wake states under non-invasive, stress-free conditions is of particular relevance for zoological institutions, aquariums, sanctuaries, and conservation programs. Sleep quality is a key indicator of animal welfare, with inadequate sleep negatively impacting cognitive, immune, and metabolic functions (4). By enabling routine EEG monitoring, this methodology provides an objective tool for assessing welfare in captive and managed animals.

Furthermore, this non-invasive methodology also demonstrates immediate and transformative potential for clinical veterinary practice. From a diagnostic perspective, it enables routine neurological screenings without the need for sedation, thereby facilitating the early detection of epilepsy, sleep disorders, age-related cognitive decline, encephalitis, and intracranial neoplasms (37, 35). For therapeutic monitoring—particularly in epileptic animals (both human and non-human)—it provides an objective means to assess treatment efficacy through serial recordings documenting seizure frequency and severity. In zoological and wildlife medicine, where anesthesia poses considerable risk, this technique allows for the evaluation of neurological function without compromising animal welfare. Furthermore, the relatively low cost of the device compared to conventional veterinary EEG systems, together with the simplicity of the recording procedures, democratizes access to advanced neurophysiological assessments. Such accessibility has the potential to markedly enhance standards of veterinary neurological care by enabling earlier and more accurate diagnosis, as well as more effective follow-up of therapeutic interventions (38). Its potential applications extend also to agricultural and domestic contexts where sleep quality may influence productivity and health (20).

### Limitations and future directions

While the methodology proved effective, certain limitations should be acknowledged. Variability in signal amplitude was observed across species, likely reflecting anatomical differences such as skull thickness and integumentary structures (20, 39). Although these differences did not preclude identification of sleep–wake states, further optimization of electrode placement may improve signal consistency (40). Additionally, aquatic species such as penguins could not be monitored in water due to the technical limitations of the current device. Future developments should focus on waterproofing and expanding the range of wireless transmission, enabling full ecological validity in aquatic environments (41). Finally, while this study demonstrated the feasibility of non-invasive EEG across three species, broader application to other taxonomic groups is necessary to establish its generalizability and refine species-specific protocols (42).

### Conclusions of the discussion

Overall, this study provides the first demonstration of a non-invasive and wireless EEG methodology capable of recording naturalistic sleep–wake cycles in unanesthetized, unrestrained reptiles, birds, and mammals. The technique represents an important step forward for comparative neurobiology and animal welfare, offering a reproducible, welfare-compatible approach that bridges technological innovation with biological and veterinary applications.

## Conclusion

This study demonstrates that high-quality EEG recordings can be obtained in reptiles, birds, and mammals using a non-invasive methodology that respects animal welfare principles. The successful identification of wakefulness and sleep patterns across the three employed species validates both the developed technology and the applied protocol, opening new avenues for longitudinal studies of behavior, neurological pathologies, and sleep–wake cycles in animals under human care.

Although limitations exist in terms of the number of electrodes and recording duration, the presented methodology represents a significant advance toward more ethical, less invasive approaches in animal neurophysiology research. It allows the collection of valuable neurophysiological data while maintaining the welfare of the study subjects.

While we recognize that invasive methods offer higher spatial resolution and access to deep brain structures, our results demonstrate that surface electrodes are sufficient for studies of sleep–wake staging and neurological screening. This approach preserves natural behavior, minimizes stress, and provides data in ecologically valid conditions.

By integrating the principles of animal welfare, future research should adopt this framework to develop more detailed and species-specific studies of sleep–wake cycles. Such research will advance understanding of the phylogenetic evolution of sleep, its biological functions, and the identification of pathologies associated with neuro-electrical activity.

## Data Availability

The raw data supporting the conclusions of this article will be made available by the authors, without undue reservation.
